# Supplementary figures and images for: Pacbio Sequencing Reveals Identical Organelle Genomes between American Cranberry (Vaccinium macrocarpon Ait.) and a Wild Relative
Source: Genes (Basel). 2019 Apr 10;10(4):291. doi: 10.3390/genes10040291 (PMC6523495; doi:10.3390/genes10040291)

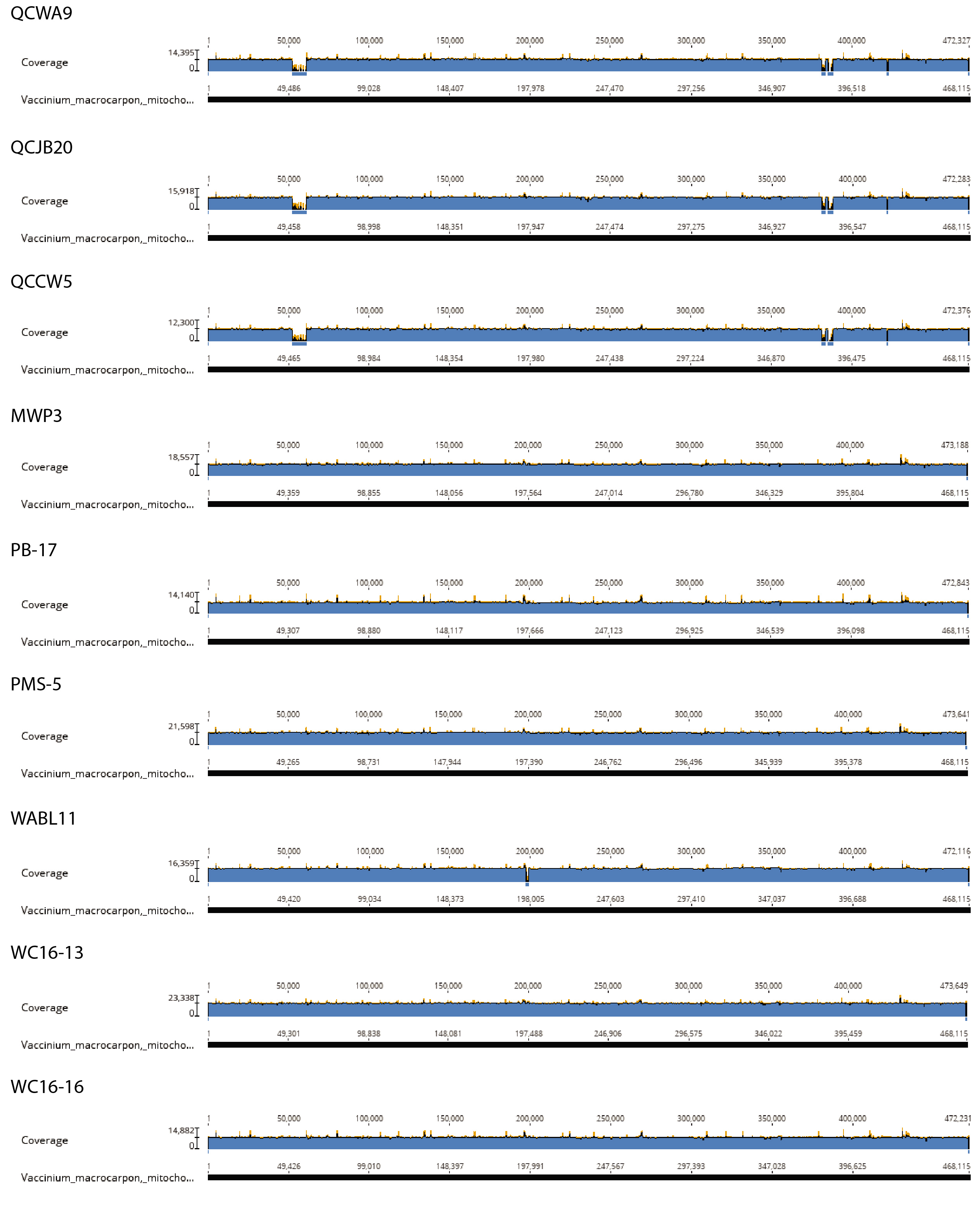

Supplement: Supplementary file 1 [file genes-10-00291-s001.zip › FigureS1.jpg]

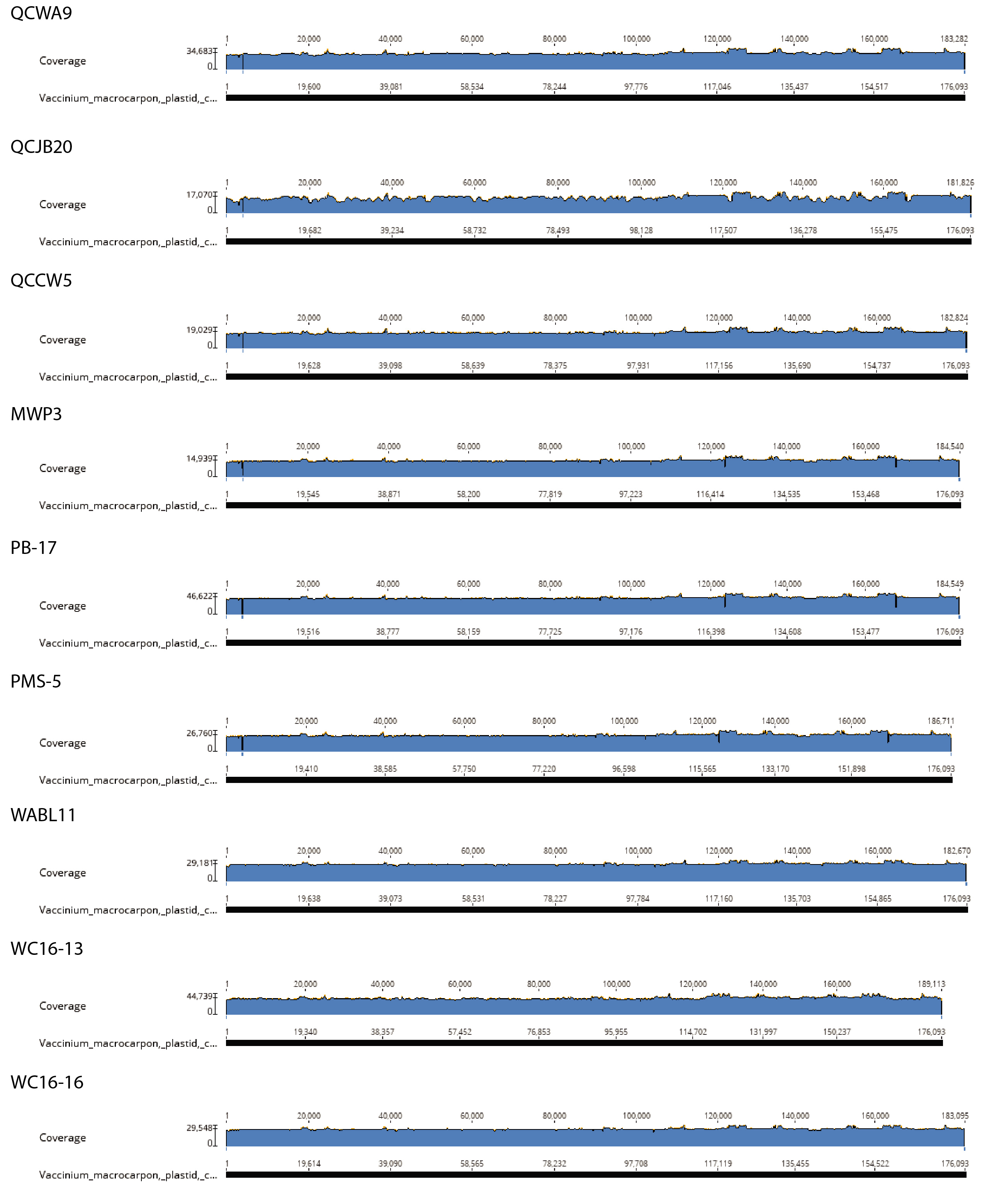

Supplement: Supplementary file 1 [file genes-10-00291-s001.zip › FigureS2.jpg]
